# An Aqueous Two-Phase System for the Concentration and Extraction of Proteins from the Interface for Detection Using the Lateral-Flow Immunoassay

**DOI:** 10.1371/journal.pone.0142654

**Published:** 2015-11-10

**Authors:** Ricky Y. T. Chiu, Alison V. Thach, Chloe M. Wu, Benjamin M. Wu, Daniel T. Kamei

**Affiliations:** 1 Department of Bioengineering, University of California, Los Angeles, Los Angeles, California, United States of America; 2 Division of Advanced Prosthodontics & Weintraub Center for Reconstructive Biotechnology, UCLA School of Dentistry, Los Angeles, California, United States of America; New York University, UNITED STATES

## Abstract

The paper-based immunoassay for point-of-care diagnostics is widely used due to its low cost and portability over traditional lab-based assays. Lateral-flow immunoassay (LFA) is the most well-established paper-based assay since it is rapid and easy to use. However, the disadvantage of LFA is its lack of sensitivity in some cases where a large sample volume is required, limiting its use as a diagnostic tool. To improve the sensitivity of LFA, we previously reported on the concentration of analytes into one of the two bulk phases of an aqueous two-phase system (ATPS) prior to detection. In this study, we preserved the advantages of LFA while significantly improving upon our previous proof-of-concept studies by employing a novel approach of concentrating gold nanoparticles, a common LFA colorimetric indicator. By conjugating specific antibodies and polymers to the surfaces of the particles, these gold nanoprobes (GNPs) were able to capture target proteins in the sample and subsequently be concentrated within 10 min at the interface of an ATPS solution comprised of polyethylene glycol, potassium phosphate, and phosphate-buffered saline. These GNPs were then extracted and applied directly to LFA. By combining this prior ATPS interface extraction with LFA, the detection limit of LFA for a model protein was improved by 100-fold from 1 ng/μL to 0.01 ng/μL. Additionally, we examined the behavior of the ATPS system in fetal bovine serum and synthetic urine to more closely approach real-world applications. Despite using more complex matrices, ATPS interface extraction still improved the detection limit by 100-fold within 15 to 25 min, demonstrating the system’s potential to be applied to patient samples.

## Introduction

Developing a detection assay for proteins that is rapid, portable, and also sensitive has been challenging in the field of diagnostics [1]–[3]. Lab-based immunoassays, such as the enzyme-linked immunosorbent assay (ELISA), display good sensitivity and are the gold standard for detecting protein targets. However, lab-based assays are not practical for use in resource-poor settings that lack power, equipment, and trained personnel. On the other hand, the paper-based lateral-flow immunoassay (LFA) is inexpensive, rapid, portable, and easy to use. However, the sensitivity of LFA is lower than that of lab-based assays, and LFA cannot therefore be used to detect target proteins that are present at low concentrations [4], [5]. Hence, while LFA is very popular and effective in detecting the glycoprotein human chorionic gonadotropin (hCG), a biomarker for pregnancy which exists abundantly in urine from a pregnant woman [6], LFA is not widely used in areas where the target proteins in sample solutions are not as abundant, such as in the detection of infectious and biowarfare agents [3], [7], [8].

The detection limit of LFA is typically 1–2 orders of magnitude higher than ELISA [2]. While concentrating targets in a sample prior to detection can improve the detection limit, concentrating proteins generally requires lab-based equipment and therefore typically cannot be combined with point-of-care assays. Our laboratory however has been focusing on concentrating the target analytes into one of the bulk phases (top or bottom) of aqueous two-phase systems (ATPSs).

The ATPS is adaptable to a practical, clinical laboratory test since it is also portable, easy to use, and phase separation does not require laboratory equipment. Some ATPSs like the polyethylene glycol (PEG)-salt system exhibit a homogeneous, isotropic phase at low temperatures, but upon increasing temperature phase separation is induced [9]. If biomolecules are present in an ATPS solution, they will distribute, or partition, between the two bulk phases based on their physical and chemical properties, such as size and hydrophobicity.

We previously concentrated biomolecules by adjusting the operating conditions of the ATPS to establish a volume ratio, defined as the ratio of the volume of the top phase to that of the bottom phase, that was much greater or much less than 1. This reduced the volume of the phase where the target molecules partitioned, effectively concentrating the target molecules in a small volume phase that was then extracted and applied to the subsequent detection assay. Specifically, we successfully used micellar and PEG-salt ATPSs to concentrate a model virus by 10-fold and subsequently improved the detection limit of LFA by 10-fold [9], [10]. For protein biomarkers, which are smaller than viruses and thus require the use of different concentration techniques, we captured the protein of interest in the sample using gold nanoprobes (GNPs), or gold nanoparticles decorated with specific antibodies. The large size of the GNPs was then used to concentrate the model protein by 10-fold, which improved the detection limit of LFA by 10-fold [11], [12].

**Fig 1:**
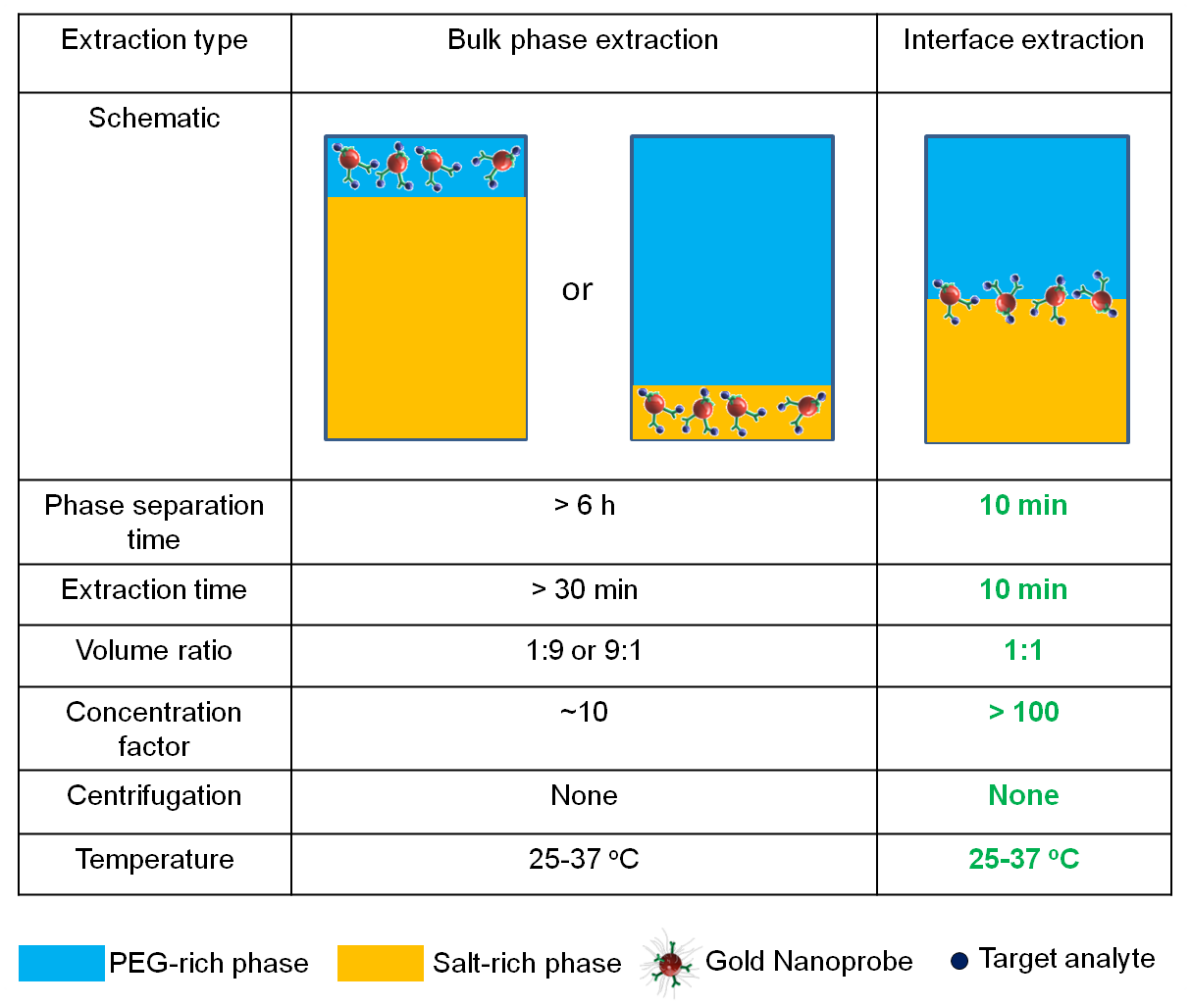
Summary of the technical innovation of engineering particles capable of partitioning to the interface of an ATPS to concentrate a target and the improvements in PBS relative to our previous proof-of-concept studies.

While we have demonstrated the combination of ATPS with LFA, the improvement of LFA depends on the fold-concentration that can be achieved in the ATPS, and this depends entirely on how small of a volume can be achieved for the target-rich phase. A more extreme volume ratio therefore will yield a more concentrated target biomolecule. A higher concentration of biomolecules will ensure that true results are obtained for the LFA competition assay format unlike the increased possibility of false negative results for the sandwich assay format as described by the hook effect [13]. However, more extreme volume ratios result in longer phase separation times since it takes longer for the microscopic domains that form the smaller phase to find each other, coalesce, and travel to the respective top or bottom phase [14]. In this study, we optimized the concentration of biomolecules using a single ATPS step by driving the target biomolecules towards the interface between the two bulk phases. Since the interfacial region represents a very small volume region that can form irrespective of the volume ratio, this novel approach allows us to concentrate the targets without dependence on extreme volume ratios, which have long phase separation times. Instead, the volume ratio that can reach equilibrium the fastest was chosen, and this reduced the extraction time to within 10 min in phosphate-buffered saline (PBS), a significant improvement over our previous approach. We also view this approach as moving towards the maximum fold-concentration that can be achieved in a single ATPS step since the volume of the interface is much smaller than the two macroscopic bulk phases. Last but not least, increasing the sample volume would increase the total number of target molecules, and would potentially lead to saturation of the antibodies for a given fixed amount of GNPs. However, the volume that can flow through the LFA test strip is limited by the size of the strip. Interface extraction allows for the sample volume to be increased without increasing phase separation time in order to detect low concentrations of target proteins, improving the sensitivity of an assay. Fig 1 pictorially compares interface extraction with extraction of one of the two bulk phases.

The technological innovation described in this manuscript is the development of nanoprobes that can localize at the interface and also serve as the colorimetric indicator for LFA. We investigated the volume ratio that phase separated the fastest and also allowed for the greatest recovery of GNPs. Subsequently, using a model protein transferrin (Tf), we demonstrated that our novel method of combining LFA with the ATPS interface extraction step is an effective yet rapid approach by improving the detection limit for LFA for Tf by 100-fold. We then extended our studies to more closely approach real-world applications, and reoptimized the system for fetal bovine serum (FBS) [15], [16] and synthetic urine [17], [18], in smaller volumes, which are preferable for blood sampling. Our data shows that, even in the more complex systems which required a few procedural modifications such as increasing the incubation time allotted for phase separation to occur, ATPS interface extraction can be performed within 15–25 min to concentrate the target 100-fold. This led to a 100-fold improvement in the detection limit of LFA for Tf, which allowed us to detect concentrations as low as 0.01 ng/μL, closing the gap in sensitivity between lab-based and paper-based immunoassays. An improved LFA with increased sensitivity would improve point-of-care solutions that require concentration of the target ligand. Overall, the ATPS interface extraction protocol is a general pre-concentration technique applicable to LFA and other detection methods when the concentration of targets is low.

## Materials and Methods

### Radiolabeling the anti-Tf antibody

All reagents and materials were purchased from Sigma-Aldrich (St. Louis, MO) unless noted otherwise. Iodine-125 (^125^I) was used to radiolabel the tyrosine residues of goat anti-human Tf polyclonal antibody (Catalog # A80-128A, Bethyl Laboratories, Montgomery, TX). Briefly, Na^125^I (MP Biomedicals, Irvine, CA) was activated by IODO-BEADS (Pierce Biotechnology, Rockford, IL). Subsequently, the activated ^125^I was reacted with goat anti-Tf antibodies for 15 min. The radiolabeled proteins were purified, and free ^125^I was removed using a Sephadex G10 size-exclusion column. The phosphotungstic acid assay was used to quantify the radioactivity and concentration of the radiolabeled proteins.

### Preparing GNPs

**Fig 2:**
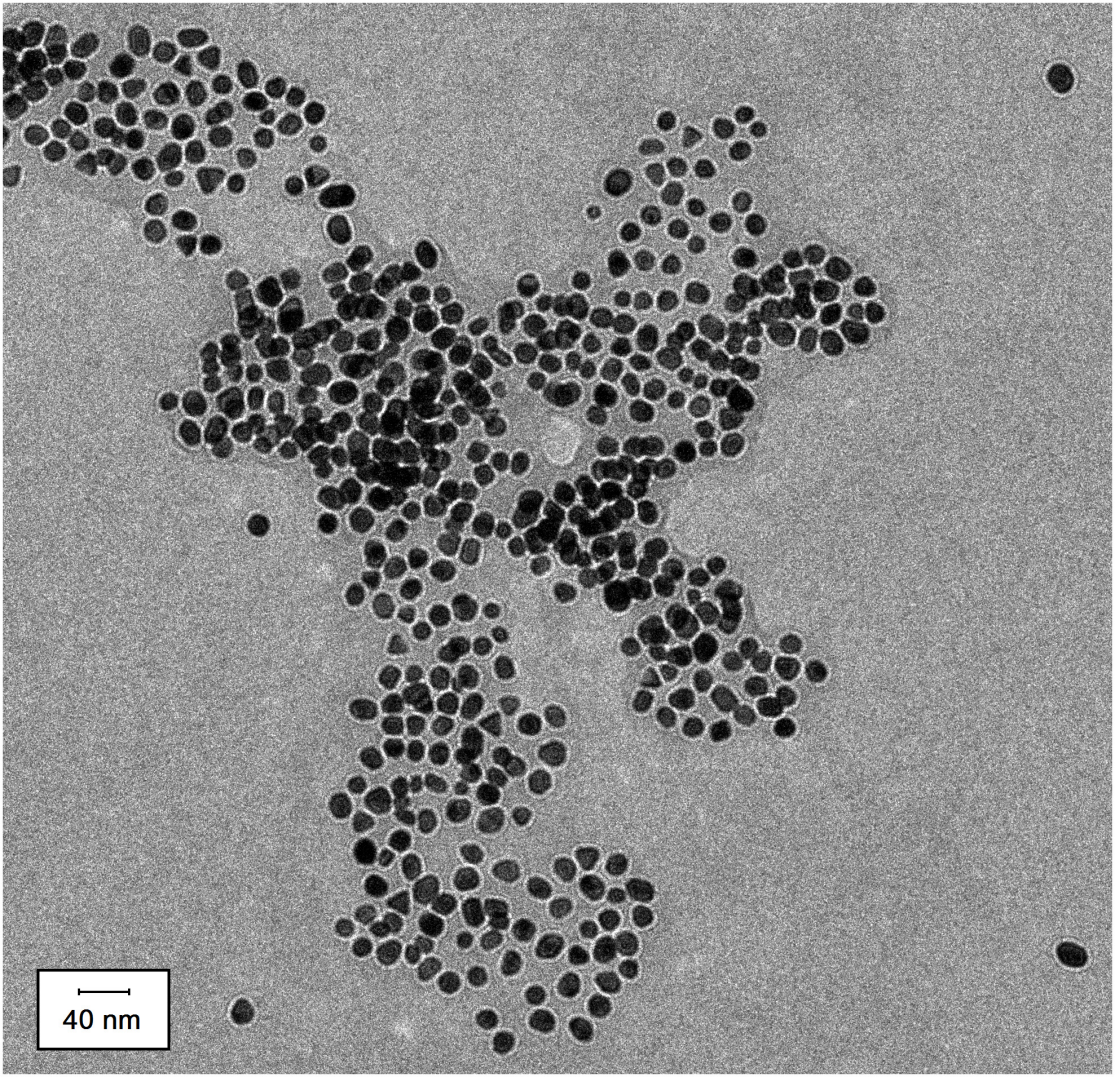
Transition electron microscopy (TEM) image of naked gold nanoparticles. Nanoparticles were suspended in filtered ultrapure water. Length of the scale bar corresponds to 40 nm. ImageJ analysis indicated the particle diameter to be 20.0 ± 3.0 nm (n = 275).

The naked gold nanoparticles were prepared using a protocol described by Frens [19], [20], resulting in a clear, cherry-colored solution with particle sizes approximately 20 nm in diameter, measured using transmission electron microscopy (TEM). Specifically, 27 mg of sodium citrate was added to 50 mL of filtered ultrapure water and 500 μL of 1% gold (III) chloride that was maintained at 100°C while stirring at 400 revolutions per minute for 2 min. The absorption (A) wavelength of the maximum Plasmon peak of the gold particles was found using a UV-Vis spectrophotometer. The diameter size of the particles was found using dynamic light scattering and was compared to a molar decadic extinction coefficient (ε) chart provided by BBInternational Life Sciences (S1 Table) to determine the corresponding ε value. For a path length (l) of 1 cm, we were able to calculate the molar concentration (C) of the gold particles by rearranging Beer’s law (*A* = *εlC*). TEM was then used to image the naked gold nanoparticles (Fig 2). 2.5 μL of the sample was placed on an EMS carbon film 200 mesh grid (Electron Microscopy Sciences, Hatfield, Pennsylvania) and filter paper was used to wick away any excess. The grid was left to air dry at ambient temperature prior to being imaged using a FEI TF20 transmission electron microscope (FEI Company, Hillsboro, Oregon) at 200 kV. The average size of the gold nanoparticles was found to be 20.0 ± 3.0 nm from analysis of the TEM image in Fig 2 using ImageJ.

To prepare the GNPs, 320 mg of goat anti-Tf antibody was incubated with 3.60 x 10^18^ colloidal gold particles, prepared in an NaOH buffer adjusted to pH 9, for 30 min, followed by the addition of 0.1 mg/mL thiolated-PEG_5000_, using a molar ratio of 3500:1 for PEG:GNP and an additional incubation of 30 min. To prevent nonspecific binding of other proteins to the surfaces of the colloidal gold, 2 mL of a 10% bovine serum albumin (BSA, Sigma Aldrich catalog #B4287, lyophilized crystal form dissolved in filtered ultrapure water) solution was added to the mixture and mixed for an additional 10 min. The resulting solution was gently mixed on a shaker during the incubation period. To remove free (unbound) antibodies, PEG, and BSA, the mixture was subsequently centrifuged for 30 min at 4°C and 9,000 g. The pellet of GNPs was washed twice with a 1% BSA solution. Finally, the recovered GNPs were resuspended in 2 mL of a 0.1 M sodium borate buffer at pH 9.0.

### Partitioning GNPs

The GNPs decorated with radiolabeled anti-Tf antibodies were partitioned in the ATPS at the different conditions shown in Table 1 to determine the volume ratio that could yield the fastest and highest GNP recovery. For each partitioning experiment, 3 identical PEG-salt solutions in Dulbecco’s phosphate-buffered saline (PBS; Invitrogen, pH 7.4, ionic strength 154 mM) were prepared to a total volume of 1500 μL. PEG-salt ATPS solutions with three different volume ratios (1:1, 6:1 and 1:6) were prepared using specific concentrations of PEG and potassium phosphate. Subsequently, 10 μL of GNP decorated with radiolabeled anti-Tf antibodies were added to each ATPS solution. The solutions were equilibrated at 0°C to ensure that the solutions were homogeneous. Once equilibrium at 0°C was attained, the solutions were incubated in a water bath at 37°C to induce phase separation, and the GNPs were found to partition between the two coexisting phases. The GNPs at the interface were withdrawn carefully using a pipette, and 30 μL of the interface solution were withdrawn to ensure most, if not all, of the GNPs at the interface were collected. The two coexisting phases were also completely withdrawn separately using pipettes. The amounts of GNPs at the interface and in the two coexisting phases were quantified by measuring the amount of radioactivity in each region using the Cobra Series Auto-Gamma Counter since the GNPs were bound to radiolabeled anti-Tf antibodies. The quantified amount of GNPs in each of the three regions was used to calculate the recovery percentage of the GNPs at the interface using a mass balance equation.

**Table 1:** Recovery of the GNPs as a function of different phase volume ratios.^a^

	Volume ratio (top phase:bottom phase)		
	1:1	6:1	1:6
**Phase separation time (min)**	10	60	30
**Radioactivity of GNP at interface (cpm ± SD)**	4130 ± 290	3240 ± 200	2390 ± 520
**GNP recovery at interface (%)**	84.1 ± 1.8	64.8 ± 1.8	70.9 ± 6.6
**Radioactivity of GNP in top phase (cpm ± SD)**	371 ± 80	940 ± 71	547 ± 140
**GNP recovery in top phase (%)**	7.5 ± 1.3	18.8 ± 1.5	16.1 ± 0.9
**Radioactivity of GNP in bottom phase (cpm ± SD)**	408 ± 61	818 ± 110	450 ± 180
**GNP recovery in bottom phase (%)**	8.3 ± 1.1	16.3 ± 1.8	13.0 ± 3.2

^a^Studies were performed using 1.5 mL of ATPS. Data are reported as mean ± standard deviation (SD), where n = 3.

### Preparing the LFA test strip

**Fig 3:**
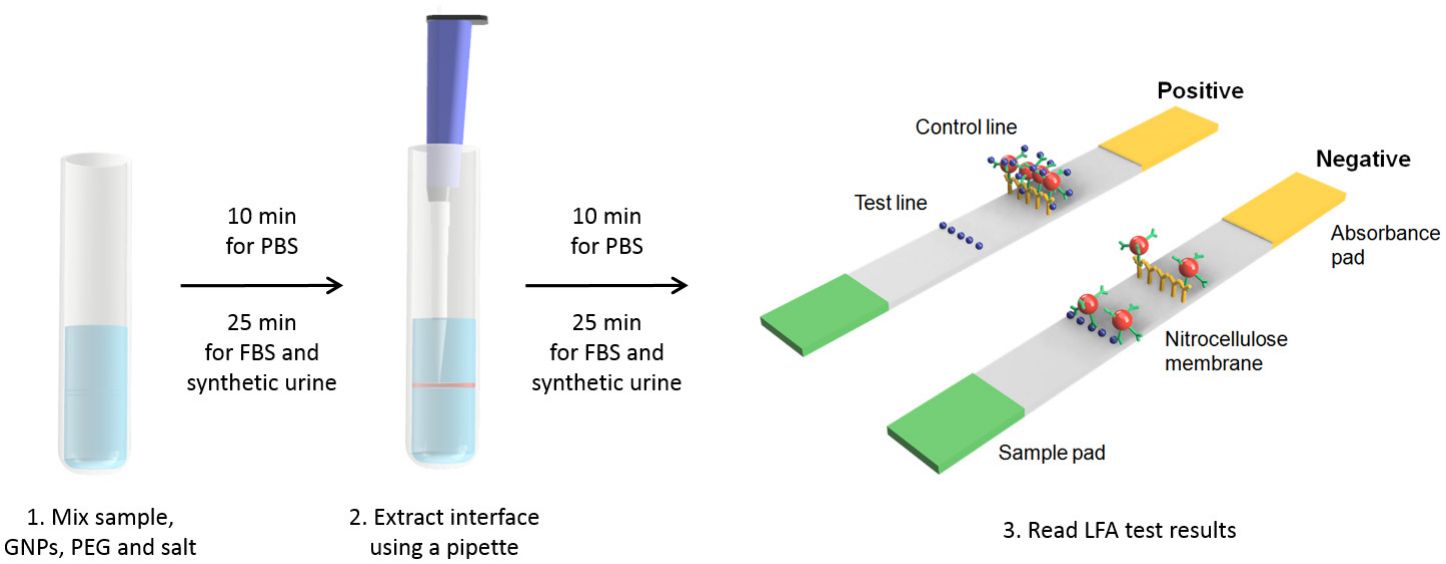
Schematic representation of the integration of ATPS interface extraction with competition-based LFA and the interpretations of the positive and negative results. An ATPS solution was constructed and allowed to phase separate for 10 min in PBS and 25 min in FBS and synthetic urine in a glass tube prior to the extraction of 30 μL of the interface containing GNPs. The extracted sample was then applied to an LFA test strip and results were read after 10 min for the PBS system and after 25 min for the FBS and synthetic urine systems. The appearance of only the control line indicated a positive result while the appearance of both the control and test lines indicated a negative result.

A competition mechanism was implemented for the LFA (Fig 3). In the competition assay [12], the target of interest is immobilized on a nitrocellulose membrane to form the test line. Immobilized secondary antibodies against the primary antibodies on the GNPs make up the control line. The antibodies on the GNPs will always bind to the immobilized secondary antibodies, creating a visible control line which indicates a valid test. The GNPs form a visual band at the test line if their conjugated antibodies are not saturated with the target. As shown in Fig 3, a positive result will be indicated by the presence of one visual band while a negative result will be indicated by the presence of two visual bands.

The lateral flow strip consists of a nitrocellulose membrane, as well as cellulose paper for the sample pad and absorbance pad. 50 μL sucrose solutions were prepared to be printed across the nitrocellulose membrane using a Becton Dickinson plasti-pak syringe and a Harvard Apparatus PHD 2000 microfluidic syringe pump set at an infuse rate of 250 μL/min. The control line was printed using a 0.5 mg/mL solution of anti-goat IgG (Bethyl Laboratories). The test line was printed using a 2.5 mg/mL solution of Tf. The three paper components of the lateral flow strip were connected through an adhesive backing.

### Performing LFA with Tf but without pre-concentration

Tf stock solutions containing varying concentrations of Tf were prepared in PBS. Subsequently, 20 μL of each Tf stock solution were added to 10 μL of the GNP suspension and 20 μL of test buffer (0.2% BSA, 0.3% Tween20, 0.2% sodium azide, 0.1% PEG, 0.1 M Trizma buffer, pH 8), which were used to aid the flow of the samples through the test strips. A total of 5 sample solutions (50 μL each) with various concentrations of Tf were prepared (0 (negative control), 0.001, 0.01, 0.1, and 1 ng/μL). A test strip was dipped vertically into each sample solution, where the sample pad would come in contact with the solution. After 10 min, the test strips were taken out, and an image of each strip was immediately taken by a Canon EOS 1000D camera (Canon U.S.A., Inc., Lake Success, NY).

In the experiments performed in FBS (HyClone, characterized, pH 7.4), a 270.6 ng/mL GNP suspension was used so that the volume of GNP could be scaled down appropriately for the lower-volume experiments. The concentrations of Tf in the FBS stock solutions were adjusted to achieve the same final Tf concentrations used in the PBS experiments by adding 5 μL of a Tf stock solution to 5 μL of GNP suspension, followed by 40 μL of test buffer. Similarly, experiments were conducted using synthetic urine prepared with a method described by Martinez [21].

### Combining the ATPS interface extraction with LFA for Tf

A volume ratio of 1:1 was used for the study conducted in PBS based on the findings from the *Partitioning GNPs* experiment. By utilizing anti-Tf antibodies, the GNPs first captured Tf in the sample, followed by the entire Tf-GNP complex being concentrated at the interface. A similar protocol to that described in the *Partitioning GNPs* section was used except that various concentrations of Tf were also spiked into the ATPS solutions. Briefly, 10 μL of the 69.7 ng/mL GNP suspension were added to 4990 μL of the Tf-spiked ATPS solution that yielded a 1:1 volume ratio and that contained various Tf concentrations (0 (negative control), 0.001, 0.01, and 0.1 ng/μL). The solutions were equilibrated at 0°C to ensure that the solutions were homogeneous. Once equilibrium was attained, the solutions were placed in a water bath at 37°C to trigger phase separation. After 10 min, 30 μL of the interface solution, which contained the Tf and the 19 ng/mL concentrated GNPs that were concentrated approximately 42-fold, were withdrawn. This interface solution was mixed with 20 μL of test buffer to form the 50 μL sample solutions. The lateral flow strip was inserted vertically into a tube containing the solution, and the tube container held the strip. After 10 min, the test strips were taken out, and an image of each strip was immediately taken by a Canon EOS 1000D camera.

For the studies conducted in FBS and synthetic urine, the PEG and potassium phosphate concentrations needed to first be adjusted to achieve the 1:1 volume ratio. The ATPS in FBS also phase separated more slowly, and instead of the 10 min incubation used in the PBS system, the solutions were kept in a 37°C water bath for 25 min. In addition, as mentioned earlier, the volumes were reduced to more closely resemble a practical application. Therefore, rather than show a detection limit increase using 5000 μL (100 times more volume than the 50 μL Tf stock solution used in the LFA only experiments), the studies performed in FBS and synthetic urine showed an equivalent improvement using 1000 μL (100 times more volume than the 10 μL Tf stock solution used in the LFA only experiments). The protocol previously described for PBS was modified for the lower volumes, so that 5 μL of the more concentrated gold suspension were added to 995 μL of the Tf-spiked ATPS solution in FBS or synthetic urine. 20 μL of the interfacial region were extracted, followed by the addition of 30 μL test buffer. Each LFA strip was dipped in the suspension for 15 min before being taken out and imaged.

### Quantitative Analysis of LFA Results

The images taken of the LFA test strips were analyzed using a custom MATLAB script. To quantify the line intensities of our results, the images were cropped and converted to 8-bit grayscale matrices. These matrices were split in half in order to produce one matrix containing the control line and the other containing the test line. Each matrix was then analyzed separately to determine the location of the control or test line by identifying the darkest spot with minimum intensity using vectors perpendicular to the line of interest. The average location of the minima found was centered on a 15 pixel-high rectangular region that spanned the length of the control and test lines, where the average grayscale intensities were denoted as *I*_*control*_ and *I*_*test*_, respectively. In order to normalize the intensities of the control and test lines, the average grayscale intensity of a reference region, denoted as *I*_*reference*_, was used to remove the effects of any background color present. The reference region was defined to be 15 pixels wide and 50 pixels upstream from the test line. Signal intensities of the control and test lines were then found using the following equations:

$${\text{Signal}}_{\text{control }}={\text{ I}}_{\text{reference}}-{\text{I}}_{\text{control}}$$

$${\text{Signal}}_{\text{test }}={\text{ I}}_{\text{reference}}-{\text{I}}_{\text{test}}$$

Signal intensity of each test line was then converted to relative test signal intensity through division by the maximum test signal intensity in the corresponding set of images. Plots of relative test signal intensity versus transferrin concentration were then made.

## Results and Discussion

### Engineering of the GNPs for Optimal Interface Partitioning

**Fig 4:**
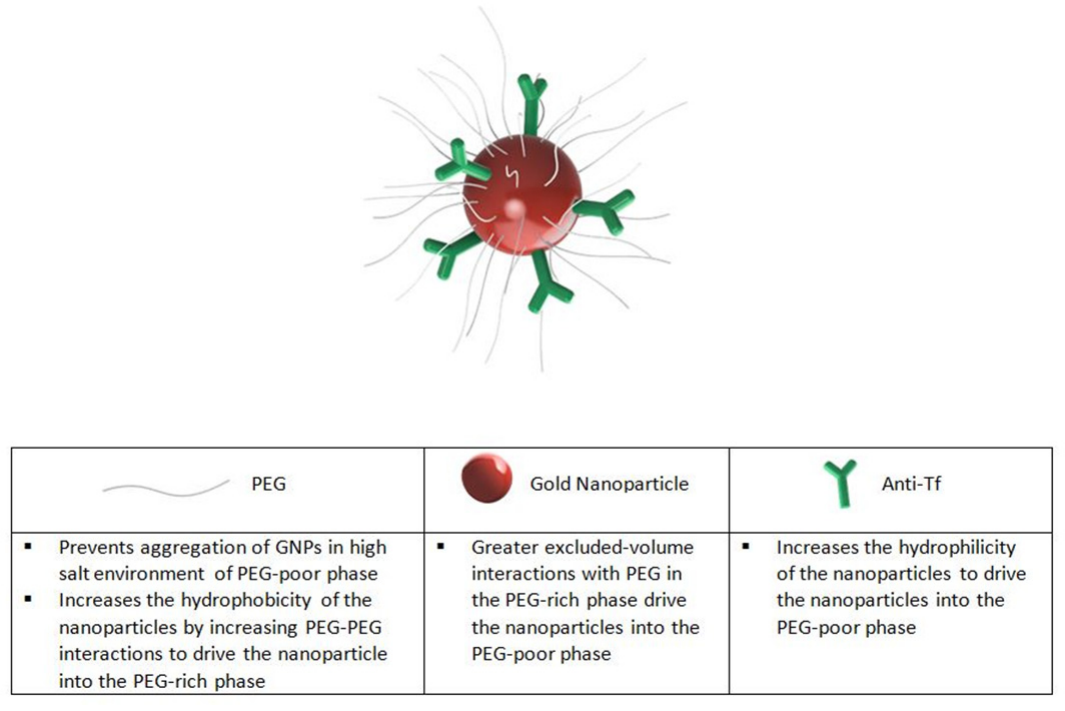
Surface modification of GNP to influence partitioning behavior in ATPS. Schematic of GNP and the functionality of each component.

**Fig 5:**
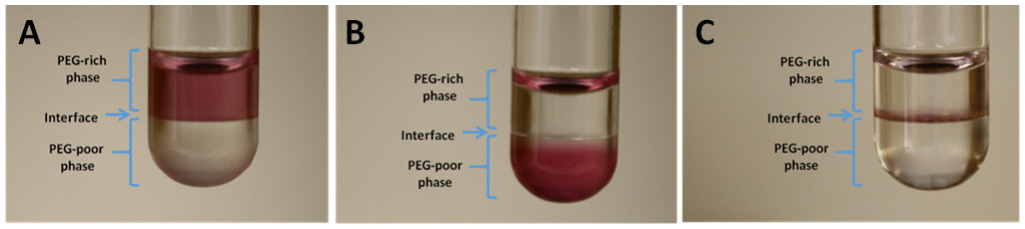
Demonstration of the partitioning behavior of GNPs in our PEG-salt ATPS. Various amounts of PEG were conjugated to the GNPs to manipulate their partitioning behavior: (A) Using a molar ratio of 5800:1 PEG:GNP during conjugation, the resulting GNPs partitioned preferentially into the PEG-rich top phase. (B) Using a molar ratio of 1200:1 PEG:GNP during conjugation, the GNPs partitioned into the PEG-poor bottom phase. (C) Using a molar ratio of 3500:1 PEG:GNP during conjugation, the resulting GNPs partitioned exclusively to the interface. For (A), (B), and (C), the red observed at the very top of the liquid-air interface was due to a reflection and not due to the presence of nanoprobes. Studies were performed in glass tubes 12 x 75 mm in size.

In order to combine the ATPS interface extraction with the paper-based LFA detection assay, the GNPs developed in this study possessed three functions. First, the decorated specific antibodies on the surfaces of the GNPs captured the target proteins present in the sample. Second, the optimized formulation of PEG and proteins on the surfaces of the GNPs caused the GNPs to partition to the interface and not the bulk phases. Lastly, the GNPs acted directly as the colorimetric indicator for LFA, and hence allowed the subsequent detection assay to be performed immediately without extra washing or other preparation steps. A schematic of the GNP is shown in Fig 4. The GNP has 3 main components: the PEG polymers, the gold nanoparticle, and the anti-Tf antibodies. Each component by itself would drive the nanoparticle into one of the two bulk phases. First, decorated PEG drives the nanoparticle into the top PEG-rich phase due to the favorable PEG-PEG interactions between the polymer on the particle surface and the abundant polymers in the top phase (Fig 5A). Specifically, increasing the molar ratio of PEG:GNP changes the conformation of the bound PEG to more closely resemble a “brush” conformation, expanding the amount of surface area exposed to increase PEG-PEG interactions [22]. On the other hand, the large size of the gold nanoparticle causes the nanoparticle to partition into the bottom PEG-poor phase where it experiences fewer repulsive, excluded-volume interactions with the PEG polymers. The hydrophilic proteins (anti-Tf Ab and BSA) on the GNP increase the hydrophilicity of the GNP, and also cause it to partition into the bottom PEG-poor phase, which is more hydrophilic than the top PEG-rich phase (Fig 5B). In combination, the 3 components of the GNP can be varied and delicately balanced to ultimately drive the GNP to the interface in our ATPS (Fig 5C).

### Identifying the Optimal Volume Ratio

Three volume ratios were tested to determine the optimal volume ratio that could recover the most GNPs within the shortest period of incubation. The results are shown in Table 1. It is not surprising to observe that the 1:1 volume ratio phase separated the fastest and allowed for the greatest recovery of the GNPs. When phase separation is triggered by increasing the temperature, microscopic PEG-rich and PEG-poor domains are formed, and similar domains will find each other and coalesce. As the domains coalesce, they travel and eventually form the macroscopic PEG-rich, salt-poor phase on top and the macroscopic PEG-poor, salt-rich phase on the bottom due to the interfacial tension and the density difference between the two phases. A 1:1 volume ratio phase separates faster than the 6:1 or 1:6 volume ratios since the domains have an easier time finding each other and coalescing when there is a significant amount of each phase. For more uneven volume ratios, domains of the smaller volume phase can be entrained in the larger continuous phase due to the domains experiencing difficulty coalescing. Moreover, the 6:1 volume ratio is expected to phase separate more slowly than the 1:6 volume ratio since the PEG-rich phase is the continuous phase for the 6:1 volume ratio, and the PEG-poor domains experience more difficulty finding each other and moving to their respective macroscopic phase in the more viscous PEG-rich continuous phase.

Since the GNPs do not partition into either domain, they remain between the domains as the domains coalesce. Eventually, the GNPs appear as a thin red film at the interface when phase separation is completed. The recovery of GNPs is more efficient when using the 1:1 volume ratio as entrainment is minimized at this volume ratio and less of the GNPs would therefore be lost to the interfaces that are present between the entrained domains and the continuous phase. Since the 1:1 volume ratio phase separated the fastest while yielding the highest GNP recovery, it was used in the subsequent experiments.

### Improving LFA Detection by Using Interface Extraction

**Fig 6:**
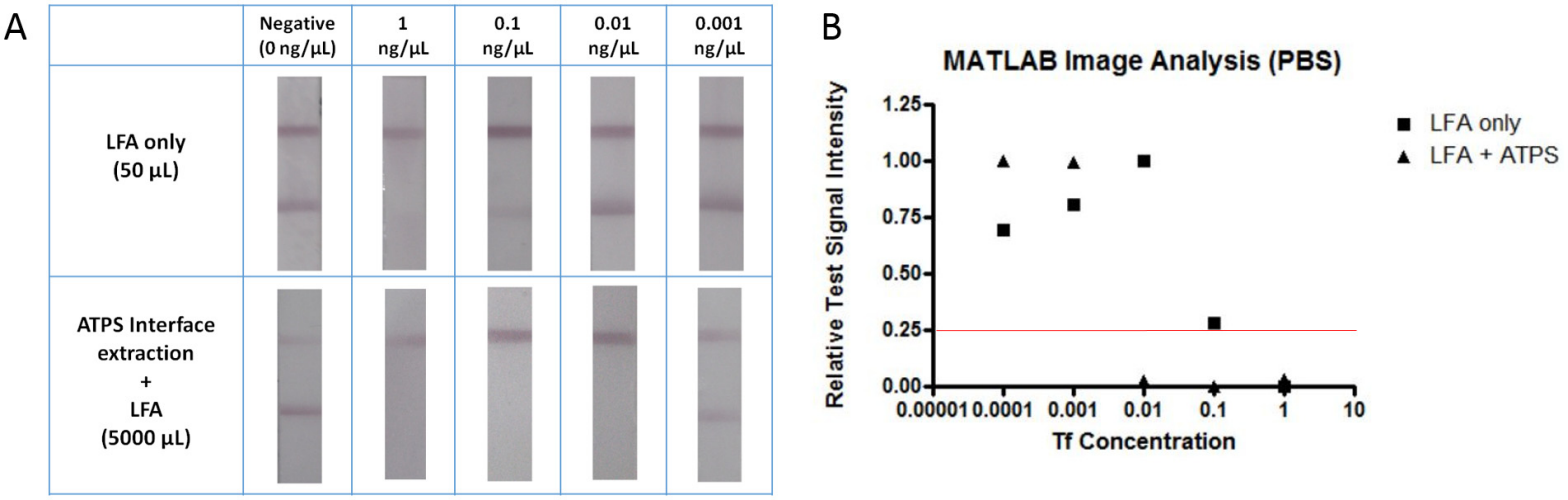
Results of LFA for detecting Tf in PBS. (A) Images of test strips without (top panel) and with (bottom panel) the prior concentration step using the ATPS interface extraction step. 50 μL sample solutions were applied to each LFA test strip. (B) MATLAB quantification of test signal intensity where a value above a threshold of 0.25 corresponded to a negative test.

To demonstrate the enhancement of LFA by incorporating the ATPS interface extraction step, we utilized the model protein transferrin (Tf). Tf is a serum protein for iron transport, and in addition to both Tf and its antibody being commercially available and inexpensive, we have experience radiolabeling the Tf antibody, which was important in determining GNP recovery. To establish the detection limit of Tf in LFA, we performed a series of LFA tests with various Tf concentrations without any prior concentration step. If a sample contained enough Tf molecules to saturate the anti-Tf antibodies decorated on GNP, then these anti-Tf antibodies did not bind to the immobilized Tf on the nitrocellulose membrane at the test line and therefore did not form a visual band at the test line. This indicated a positive result, which was observed when testing the sample with a Tf concentration of 1 ng/μL (Fig 6A, top panel). On the other hand, if insufficient or no Tf was present in the sample to saturate the anti-Tf antibodies, then these anti-Tf antibodies did successfully bind to the immobilized Tf on the nitrocellulose membrane and therefore formed a visual band at the test line. This indicated a negative result, which was observed when testing samples with Tf concentrations less than 1 ng/μL. Since 1 ng/μL is the lowest Tf concentration that showed a true positive result, this indicated a detection limit of approximately 1 ng/μL for Tf when performing LFA without the prior concentration step.

To determine if the ATPS interface extraction step could improve the detection limit of Tf by 100-fold using LFA, we applied the same amount of the GNPs to the ATPS solutions with Tf concentrations that were 100 times lower than the detection limit of LFA (0.01 ng/μL). Since we had an idea of the number of Tf molecules required to saturate the antibodies, we increased the sample volume 100-fold from 50 μL to 5000 μL to keep the total number of Tf molecules the same. Since only a limited amount of sample (50 μL) could be applied to an LFA test strip, the diluted GNPs in this larger sample solution needed to be concentrated and applied to LFA to obtain a valid result. To recover these GNPs that were saturated with the target proteins, we placed the solution in a water bath at 37°C to collect the GNPs at the interface within 10 min. The GNPs were then extracted and applied directly to the LFA test strip. The results of this study are shown in the bottom panel of Fig 6A. We were able to obtain a true positive result at 0.01 ng/μL, which showed a 100-fold improvement in the detection limit. The test line intensities of the false negative result at 0.001 ng/μL using this approach were lighter than those without the prior concentration step when comparing samples with the same Tf concentration, indicating that more Tf was captured to make it difficult for the GNPs to bind to the test lines. The test line intensities also increased as the Tf concentration decreased, which was expected as the amount of Tf available to saturate the antibodies decreased. If GNPs were lost to either of the two domains prior to interface extraction, the line intensities of the subsequent LFA test would be expected to be diminished, improving the limit of detection of the assay. However, the loss of too many GNPs would produce a control line that is too faint in intensity and the LFA test result would be invalidated.

**Fig 7:**
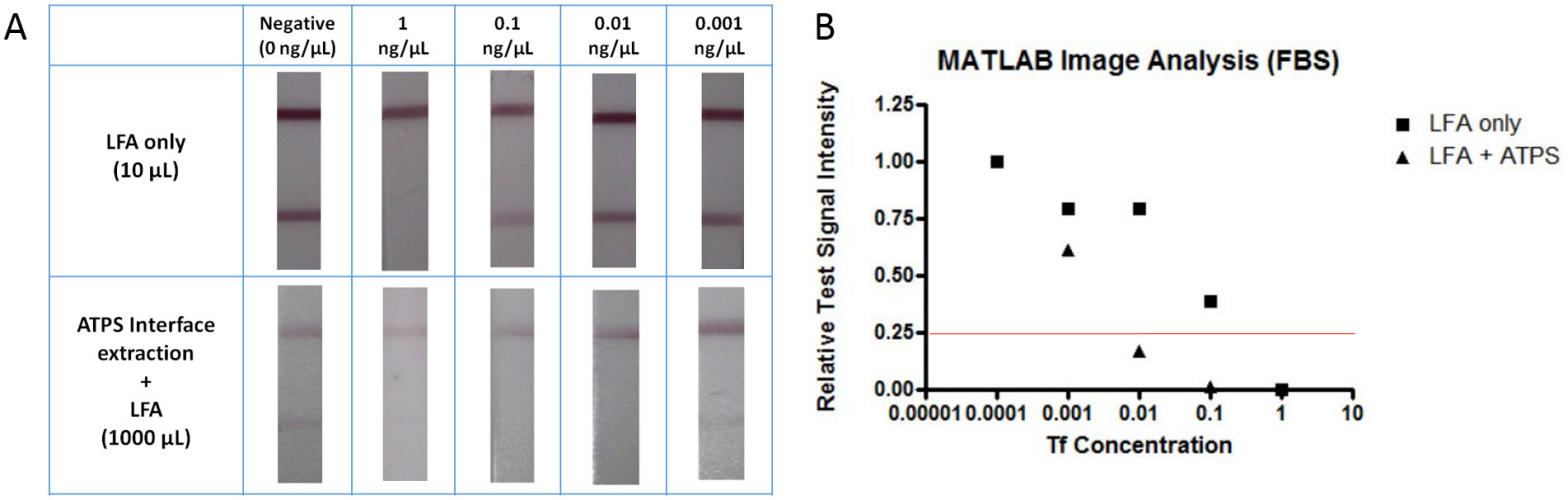
Results of LFA for detecting Tf in FBS. (A) Images of test strips without (top panel) and with (bottom panel) the prior concentration step using the ATPS interface extraction step. 50 μL sample solutions were applied to each LFA test strip. (B) MATLAB quantification of test signal intensity where a value above a threshold of 0.25 corresponded to a negative test.

**Fig 8:**
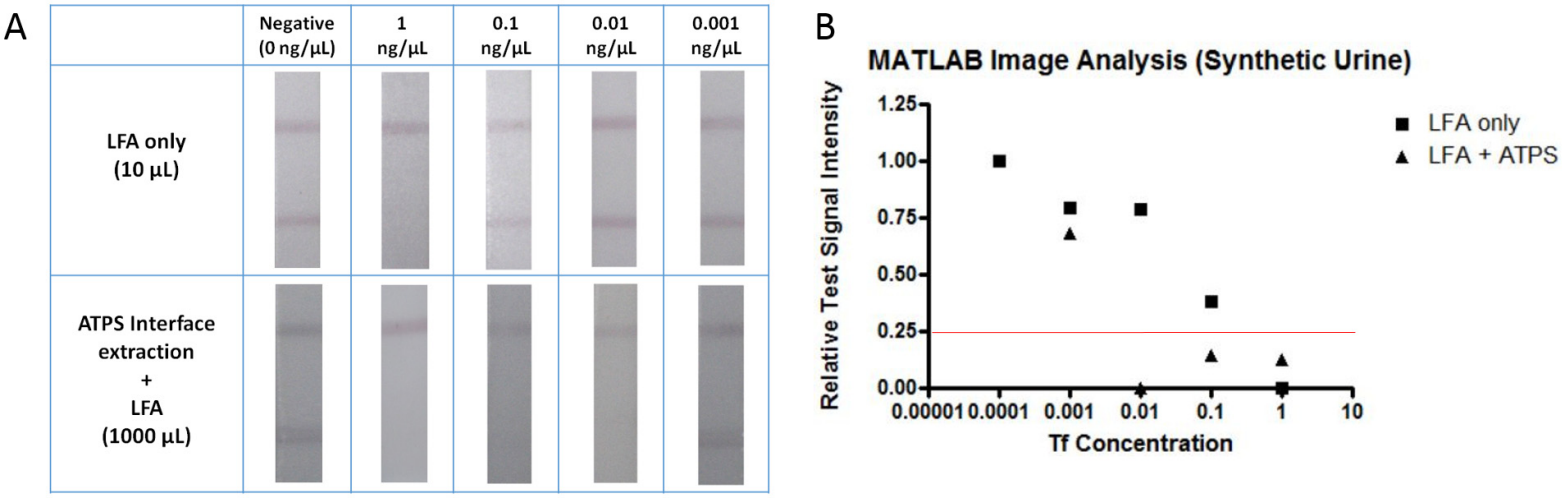
Results of LFA for detecting Tf in synthetic urine. (A) Images of test strips without (top panel) and with (bottom panel) the prior concentration step using the ATPS interface extraction step. 50 μL sample solutions were applied to each LFA test strip. (B) MATLAB quantification of test signal intensity where a value above a threshold of 0.25 corresponded to a negative test.

To study the effectiveness of ATPS interface concentration in a system more likely to be applied to a future device, we tested lower volume ATPS solutions made with FBS to mimic a small sample blood draw from a patient. Due to the more complicated composition of FBS, the procedure used with the ATPS in PBS was reoptimized for the FBS system. The higher protein content of FBS altered the volume ratio of the ATPS, requiring different concentrations of PEG and salt to form a 1:1 volume ratio. Since the experiments performed in FBS also utilized smaller sample volumes, the volume of GNP had to be scaled down, and a more concentrated GNP stock was made. In addition, the incubation time for the ATPS was extended from 10 to 25 min as the FBS slowed down the phase separation process. Additionally, due to the complex mixture comprising FBS, the time for the LFA test was extended from 10 to 15 min. Despite serum representing a more complex matrix, Fig 7A shows that LFA combined with ATPS interface extraction still yielded a 100-fold improvement in the detection limit compared to LFA without prior concentration. As previously mentioned, we used lower volumes to more closely resemble a practical application, and used 10 μL of the sample for LFA only and 1000 μL of the sample for LFA combined with ATPS interface extraction. A similar optimization process was performed for the synthetic urine system, ultimately demonstrating an analogous 100-fold improvement in detection limit, as displayed in Fig 8A.

In order to quantitatively analyze our LFA results from Figs 6A, 7A and 8A, the signal intensities of the control and test lines were assessed using a custom MATLAB script. The relative Signal_test_ intensities obtained for each LFA test strip were compared to a threshold value of 0.25 in Figs 6B, 7B and 8B, where a relative Signal_test_ value less than 0.25 indicated positive detection of Tf and a relative Signal_test_ value greater than 0.25 indicated negative detection of Tf. When quantitatively analyzing the LFA panels for PBS, FBS, and synthetic urine, the LFA only results indicated a detection of Tf at 1 ng/μL while the results of performing ATPS interface extraction along with LFA indicated a detection of Tf at 0.01 ng/μL. Therefore, the results of the quantitative analysis of the LFA test strips in Figs 6A, 7A and 8A have confirmed our visually determined 100-fold improvement in the detection limit of LFA for Tf.

## Conclusions

In this study, a novel approach to improve the performance of the LFA paper-based immunoassay was investigated. Specifically, a multi-functional nanoprobe, or the GNP, was developed and utilized to first capture target protein molecules in a sample, then concentrate preferentially to the interface of the ATPS, and finally serve as the colorimetric indicator for LFA. Different volume ratios of the PEG-salt ATPS were investigated to achieve the fastest and greatest recovery of the GNPs at the interface. A 1:1 volume ratio was found to be optimal since over 80% of the GNPs could be recovered at the interface within only 10 min in an ATPS comprised of PEG, potassium phosphate, and PBS. Using this volume ratio, we subsequently demonstrated the improved performance of detecting a model protein with LFA by combining LFA with the ATPS interface extraction step. This effectively decreased the detection limit of LFA by 100-fold in PBS, FBS, and synthetic urine. Furthermore, the 100-fold improvement in detection limit demonstrated in complex fluids indicates that this new technology is robust and may eventually be implemented successfully with patient samples. We believe that this innovation will have great impact on the emerging field of paper-based assays since we provide a rapid, inexpensive, and highly effective solution for concentrating proteins with minimal power and no need for laboratory equipment.

## Supporting Information

S1 Table Molar extinction coefficient chart. Molar extinction coefficients based on gold nanoparticle diameter at the maximum of the surface-plasmon-peak. The values below were taken from the data sheet provided by BBInternational Life Science (Madison, WI).
(DOCX)
