# Supplementary material for: An Aqueous Two-Phase System for the Concentration and Extraction of Proteins from the Interface for Detection Using the Lateral-Flow Immunoassay
Source: PLoS One. 2015 Nov 10;10(11):e0142654. doi: 10.1371/journal.pone.0142654 (PMC4640584; doi:10.1371/journal.pone.0142654)
Supplement: S1 Table — Molar extinction coefficients based on gold nanoparticle diameter at the maximum of the surface-plasmon-peak. The values below were taken from the data sheet provided by BBInternational Life Science (Madison, WI). (DOCX) [file pone.0142654.s001.docx]

**S1 Table.** **Molar extinction coefficient chart.** Molar extinction coefficients based on gold nanoparticle diameter at the maximum of the surface-plasmon-peak. The values below were taken from the data sheet provided by BBInternational Life Science (Madison, WI).

| d (nm) | ε (M^-1^cm^-1^) |
| --- | --- |
| 17 | 5.502 x 10^8^ |
| 18 | 6.644 x 10^8^ |
| 19 | 7.941 x 10^8^ |
| 20 | 9.406 x 10^8^ |
| 21 | 1.105 x 10^9^ |
| 22 | 1.288 x 10^9^ |
| 23 | 1.492 x 10^9^ |
| 24 | 1.717 x 10^9^ |
| 25 | 1.964 x 10^9^ |
